# The impact of pregnancy induced hypertension on low birth weight in Ethiopia: systematic review and meta-analysis

**DOI:** 10.1186/s13052-020-00926-0

**Published:** 2020-11-26

**Authors:** Temesgen Getaneh, Ayenew Negesse, Getenet Dessie, Melaku Desta

**Affiliations:** 1 Department of Midwifery, College of Health Science, Debre Markos University, P.O. Box 269, Debre Markos, Ethiopia; 2 Department of Human Nutrition and Food Sciences, College of Health Science, Debre Markos University, Debre Markos, Ethiopia; 3 Center of excellence in Human Nutrition, School of Human Nutrition, Food Science and Technology, Hawassa University, Hawasa, Ethiopia; 4 Department of Nursing, School of Health science, College of Medicine and Health Science, Bahr Dar University, Bahir Dar, Ethiopia

**Keywords:** Low birth weight, Pregnancy induced hypertension, Ethiopia

## Abstract

**Background:**

Even though neonatal mortality reduction is the major goal needed to be achieved by 2030, it is still unacceptably high especially in Ethiopia. In the other hand, low birth weight is the major cause of neonatal mortality and morbidity. More than 10 millions of low birth weight infants occurred as a result of pregnancy induced hypertension. However, in Ethiopia the association between low birth weight and pregnancy induced hypertension was represented with un-updated, inconclusive and different studies. Therefore, this review aimed to estimate the overall pooled impact of pregnancy induced hypertension on low birth weight and its association in Ethiopia.

**Methods:**

articles searched on PubMed/Medline, EMBASE, CINAHL, Cochrane library, Google, Google Scholar and local shelves. Joanna Briggs Institute Meta-Analysis of Statistics Assessment and Review Instrument (JBI-MAStARI) was applied for critical appraisal. The *I*^*2*^ statistic was computed to check the presence of heterogeneity. Publication bias was evaluated using funnel plot asymmetry and Egger’s test. A random effect model was used to estimate the pooled prevalence of low birth weight.

**Result:**

From the total 131 identified original articles, 25 were eligible and included for the final analysis. The overall pooled prevalence of low birth weight among women who had pregnancy induced hypertension in Ethiopia was 39.7% (95% CI: 33.3, 46.2). But, *I*^*2*^ statistic estimation evidenced significant heterogeneity across included studies (I^2^ = 89.4, *p* < 0.001). In addition, the odds of having low birth weight newborns among women who had pregnancy induced hypertension was 3.89 times higher compared to their counterparts (OR = 3.89, 95% CI: 2.66, 5.69).

**Conclusion:**

The pooled prevalence of low birth weight among women who had pregnancy induced hypertension was more than two times higher than the pooled estimate of low birth weight among all reproductive aged women. The odds of low birth weight also increased nearly four times among women with pregnancy induced hypertension than normotensive women. Therefore, health policies which provide better and quality antenatal care with more oriented on importance of early detection and management of pregnancy induced hypertension should be implemented.

## Background

According to the World Health Organization (WHO), Low birth weight (LBW) is defined as a birth weight of less than 2500 g irrespective of the gestational age. It is an important marker of maternal and fetal health and nutrition [1]. Globally, LBW is estimated that 15% of all births are LBW, representing more than 20.5 million births a year [2]. The great majority of LBW births occur in low and middle income countries [3]. Especially, Africa is home to about one quarter of all LBW newborns, sub-Saharan Africa contribute for 13% of LBW [4]. In Ethiopia, about 13% of newborns are LBW according to Ethiopian Demographic and Health Survey 2016 [5]. Nevertheless, LBW is a global concern, as some high-income countries are also faced with high rates for their contexts [6].

LBW is a significant public health problem, frequently associated with neonatal and child morbidity and mortality [7]. LBW newborn had higher risk of dying in the first 28 days of life [8]. In Ethiopia, more than 4.5% of neonatal mortality was due to LBW [9]. It is also associated with morbidities including stunting, metabolic syndrome, cardiovascular disorder, chronic renal insufficiency, diabetes mellitus and obesity [10]. LBW also showed significant effects on poor cognitive development, school performance and behavioral outcomes later in life [11, 12]. Moreover, LBW contributes to the costs to the family, society and the country as a whole. Neonatal hospital costs are high and it remains high after hospital discharge as children grow older costs continues to be high [13].

Globally, maternal nutrition, extreme maternal age, socio-economic status, multiple pregnancies, chronic medical conditions and pregnancy induced hypertension (PIH) are the common causes of LBW [14–17]. PIH is a significant public health threat and cause of neonatal morbidity and mortality especially in developing countries, accounting for more than 10 million LBW infants [18, 19]. In adding to this, women who had PIH were nearly ten times at increased risk of LBW [20].

Decreased utero-placental perfusion also significantly increases the risk of intrauterine growth restriction, preterm birth and other neonatal comorbidities (like respiratory distress syndrome, intraventricular hemorrhage) and other systemic disturbances [21, 22]. In addition, these children are more susceptible to neurodevelopmental and behavioral problems as well as chronic non-communicable disease later in life [12, 23, 24]. In Ethiopia, the burden of PIH ranges from 2.23 to 18.25% [25, 26]. So, LBW is a complex syndrome that includes preterm neonates, small for gestational age neonates and the overlap between these two situations, typically have the worst outcomes [27]. In addition, PIH also responsible for nearly one tenth of maternal mortality in middle and low income countries and one-fifth maternal mortality Ethiopia [28].

Reduction in LBW mortality have greatly contribute to the reduction in overall mortality and recognized as a public health priority. World Health Assembly Member States endorsed the target of 30% reduction in LBW globally in 2025 [29]. However, the world is still far from achieving this objective. So, reporting such a comprehensive finding, the effect of PIH on LBW, will have great role to achieve LBW reduction which was one of the major causes of neonatal morbidity and mortality. In a country like Ethiopia where striving to reduce neonatal mortality in 2030, investigating such concrete scientific evidence will be a major input for future neonatal health and outcome improvement.

In Ethiopia, several individual studies were done and reported inconsistent findings in the relationship between PIH and LBW, except one review published in 2019 which didn’t include all currently available researches on association of PIH and LBW (studies done after the previous review) [30]. Accordingly, the prevalence of LBW among PIH women ranges from 9.8% [31] to 64.4% [32]. In addition, the odds of developing LBW among women who had PIH range from 1.31 to 10.33 [20, 33]. Therefore, the aim of this systematic review and meta analysis was to estimate an updated pooled prevalence of LBW among women who had PIH and their association in Ethiopia. It is envisaged that results of this study could contribute to achieve reduction of LBW (and in turn neonatal morbidity and mortality) globally, especially in middle and low income countries including Ethiopia.

## Methodology

### Searching strategies

Initially, systematic review and meta analysis including registered protocols were searched to avoid duplication. Accordingly, there was one systematic review reported on adverse neonatal outcomes of PIH, which was not primary focused on LBW and different search strategies lead to evaluate different researches. In addition, there are studies published after the previous review published. Published articles were systematically searched using major databases: PubMed/Medline, EMBASE, CINAHL, Cochrane library, Google and Google Scholar. In addition to this, the reference lists of already identified articles, grey literature available on local shelves and institutional repositories were used to access unidentified and un-published articles. All articles published till June 11/2020 were included in this systematic review and meta analysis. All fields, MeSH terms and key terms combined with Boolean operators were used to search articles in electronics data bases and advanced PubMed search. The key terms used for searching were “infant” OR “neonatal” OR “child” AND “LBW” OR “abnormal weight” OR “birth weight” OR “very low birth weight” OR “adverse outcomes” OR “underweight” OR “birth outcome” AND “PIH” OR “preeclampsia” OR “gestational hypertension” AND “Ethiopia”. Then identified articles were exported into endnote citation manager software version X7 for Windows to exclude duplicate records. This review is reporting in accordance with the Preferred Reporting Items for Systematic Reviews and Meta-Analyses Protocols (PRISMA) checklist guidelines [34] (see Additional file 1).

### Eligibility criteria

#### Inclusion criteria

All studies conducted in all regional state and city of Ethiopia on reproductive aged women that reported; either the prevalence of newborn birth weight among women who had PIH or the association between PIH and neonatal birth or both were considered for this systematic review and meta analysis. In order to estimate the pooled prevalence, only women who had PIH were included while for estimating the association, both normotensive and hypertensive women were considered. Articles considered PIH as a general or specifically either gestational hypertension or preeclampsia or eclampsia were considered. There was no restriction applied in terms of type of PIH, language, study design, sampling technique, study area, study year and publication year.

#### Exclusion criteria

All identified studies titles and abstracts were screened for eligibility criteria of the review by reviewers independently. According to selection criteria, full texts of eligible studies were examined. Those papers which did not fully accessed at the time of our search process were excluded after contact was attempted with the principal investigator through email at least two times. Finally, after reviewing their full texts, studies which did not report our outcome of interest and studies with poor quality as per settled criteria of reviewing the articles were excluded from the final analysis.

### Data extraction

After standardized data extraction format developed according to 2014 Joanna Briggs Institute Reviewers’ Manual [35], two authors (TG and AN) extract the data using the spread sheet. The developed data extraction format include: study of region, publication year, study design and technique, type of PIH, sample size, mean age of respondent and prevalence as well as the cross tabulation of their association.

### Quality assessment

Joanna Briggs Institute Meta-Analysis of Statistics Assessment and Review Instrument (JBI-MAStARI) [36] was utilized for critical appraisal of included studies before data extraction. The criteria used for cohort studies includes: 1.study population recruited from the same population, 2.exposures measured similarly to assign people to both groups, 3.valid and reliable measurement for exposure, 4.confounding factors identified, 5.strategies to deal confounding factors, 6.the participants free of the outcome at the start of the study, 7.valid and reliable measurement for outcomes, 8.sufficient follow up time, follow up complete (or reasons to loss to follow up), 9.strategies to address incomplete follow up utilized and 10.appropriate statistical analysis used.

Whereas for case control studies includes: 1.comparable groups, 2.cases and controls matched appropriately, 3.similar criteria used for identification of cases and controls, 4.valid and reliable measurement for exposure, 5.similar exposure measurement, 6.confounding factors identified, 7.strategies to deal with confounding factors, 8.outcomes assessed in valid and reliable way, 9.the exposure period of interest long enough and 10.appropriate statistical analysis used and for cross sectional studies includes: 1.the criteria for inclusion in the sample clearly defined, 2.the study subjects and the setting described in detail, 3.valid and reliable measurement for exposure, 4.standard criteria used for measurement of the condition, 5.confounding factors identified, 6.strategies to deal with confounding factors stated, 7.the outcomes measured in a valid and reliable way and 8.appropriate statistical analysis used. Each individual paper were critically evaluated by two independent reviewers. Disagreements between the reviewers were resolved via consensus. If not third reviewer was involved to resolve inconsistencies in between the two independent reviewers. After quality assessment, studies which scored five and above were included in this review.

### Outcome measurement

The outcome of this systematic review and meta analysis was the prevalence of LBW among women who had PIH. LBW is defined as a birth weight of less than 2500 g irrespective of the gestational age.

### Data analysis

First, the extracted data computed in excel spread sheet were imported to STATA version 14 for further analysis. Forest plot was computed to show the pooled prevalence of LBW and heterogeneity. Cochran’s Q statistic with inverse variance (I^2^) were used to assess the existence of statistical heterogeneity and to quantify it. Low, moderate and high heterogeneity were considered at 25, 50 and 75% respectively (23). In addition, *p* value less than 0.05 was used to confirm the presence of heterogeneity across studies. Also, publication bias was assessed using Egger’s regression test [37] using *p* value less than 0.05 as a cut point to declare the presence of publication bias and used a funnel plot to represent graphically the bias. The estimated pooled prevalence of LBW was presented using forest plot diagram with their corresponding 95% CI and OR. Finally, subgroup analysis and meta regression were computed to explore those potential heterogeneity across studies.

## Results

### Explanation of original studies

**Fig. 1:**
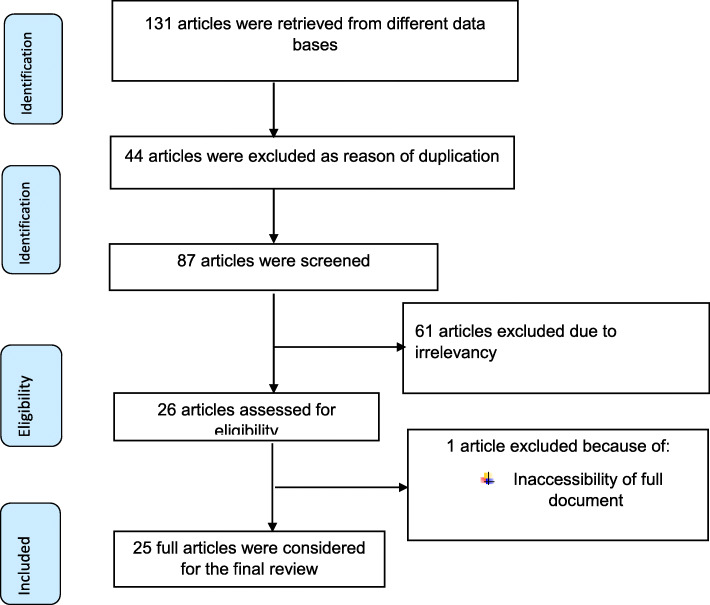
PRISMA flow diagram of included studies to estimate the effect of PIH on LBW prevalence in Ethiopia: 2005–2020

A total of 131 articles were retrieved from electronic data bases and gray literatures related to association between LBW and PIH in Ethiopia. Then, 44 articles were excluded because of duplication. From the remaining, 61 articles were removed as reason of irrelevance of titles to this review. Out of these, after reviewing the remaining studies, one article was excluded because of inaccessibility of full text (difficult to assess the quality of this paper without full document) [38]. Finally 25 articles were eligible and considered for this systematic review (Fig. 1).

Based on our inclusion criteria, only 25 articles were included and considered for final analysis. From those included studies, eight articles were done in Amhara region [20, 32, 33, 39–43], seven in SNNP [31, 44–49], four articles in AA [50–53] and Oromo [25, 54–56] for each and one article in Tigray [57] and Ethiopian Somalia region [58] for each. Concerning to publication, one study was un-published (grey literature) [40] whereas the remaining 24 articles were published from 2005 to 2020 in different journals [20, 25, 31–33, 39, 41–50, 52, 53, 55–58]. Regarding to study design, 15 articles were employed cross sectional [20, 25, 31, 39, 41–43, 47–51, 53–56] while the five studies were done using cohort [33, 45, 50, 57, 58] and case control design [32, 40, 44, 46, 52] for each studies scored five and above out of settled criteria for each study design according to JBI critical appraisal were included for this systematic review and meta analysis (Table 1).

**Table 1:** Descriptive summary of included studies in this systematic review and meta-analysis to assess association of LBW and PIH in Ethiopia

Author	Study Year	Region	Study design	Sampling technique	Mean age	Type of PIH	Sample size	Prevalence (%)	JBI score
Wagnew et al [53]	2016	AA	Cross-sectional	Consecutive	26.75	PIH	1809	44.2	6
Seyom et al [54]	2015	Oromo	Cross-sectional	Consecutive	24.4	PIH	121	31	5
Melese et al [42]	2018	Amhara	Cross-sectional	Consecutive	28.3	PIH	456	13	7
Asseffa et al [31]	2019	SNNP	Cross-sectional	Consecutive	25.4	PIH	168	9.8	6
Gudeta et al [55]	2017	Oromo	Cross-sectional	SYRS	–	PIH	33	24.2	7
Jima A et al [50]	2014	AA	Cohort	Consecutive	–	Eclampsia	78	61.5	6
Obsa et al [49]	2018	SNNP	Cross-sectional	Consecutive		PIH	225	28.8	5
Berhe et al [57]	2020	Tigray	Cohort	Consecutive	27.34	PIH	260	37.7	8
Gudu et al [58]	2018	Somalia	Cohort	Consecutive	23	Eclampsia	75	45.3	7
Wolde et al [56]	2011	Oromo	Cross-sectional	Consecutive	–	PIH	146	41.09	5
Vata et al [25]	2015	Oromo	Cross-sectional	Consecutive	–	PIH	172	26.74	6
Terefe et al [43]	2015	Amhara	Cross-sectional	Consecutive	25.4	PIH	226	39.4	6
Kebede et al [33]	2013	Amhara	Cohort	Consecutive	27.27	Eclampsia	14	28.57	6
Girma et al [51]	2018	AA	Cross-sectional	SYRS	–	PIH	105	39.04	6
Desalegn et al	Un-pu	Amhara	case control	Consecutive	–	PIH	34	47	5
Bekela et al [44]	2020	SNNP	case control	Consecutive		PIH	57	49.12	7
Adane et al [39]	2014	Amhara	Cross-sectional	Consecutive	26.2	PIH	19	36.8	6
Lake et al [48]	2019	SNNP	Cross-sectional	SYRS	25	PIH	33	48.5	6
Mulu et al [52]	2020	AA	Case control	SYRS	–	PIH	56	50	7
Ekubagewargies et al [41]	2019	Amhara	Cross-sectional	SRS	27.1	PIH	31	25.08	7
Desta et al [49]	2019	SNNP	Cohort	SYRS	26.9	PIH	64	40.62	7
Zenebe et al [20]	2014	Amhara	Cross-sectional	SYRS	25.8	PIH	28	64.28	6
Gebremariam et al [47]	2005	SNNP	Cross-sectional	SRS	–	PIH	24	62.5	5
Gebrehawarya et al [32]	2018	Amhara	Case control	Consecutive	24.5	PIH	45	64.4	7
Egata et al [46]	2019	SNNP	Case control	Consecutive	–	PIH	396	–	5

### Pooled prevalence of LBW among women who had PIH in Ethiopia

**Fig. 2:**
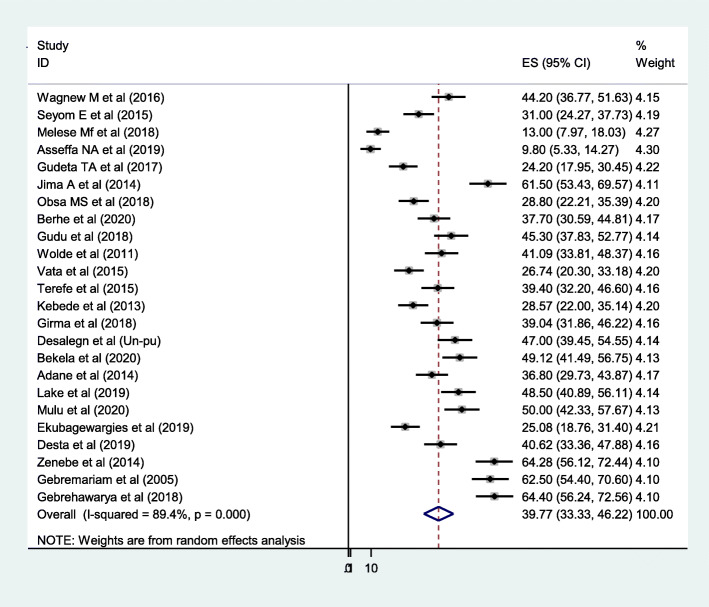
Forest plot of the pooled prevalence of LBW among women who had PIH in Ethiopia: 2005–2020

The pooled prevalence LBW among women who had PIH in Ethiopia was range from 33.3 to 46.2% whereas the point estimate of the pooled prevalence of LBW among PIH women was 39.77% (95% CI: 33.33, 46.22). In this review, *I*^*2*^ test statistics showed a significant level of heterogeneity across included studies (*I*^*2*^ = 89.4%, *p* < 0.001) (Fig. 2). Therefore, to estimate the pooled prevalence of LBW and its association between LBW and PIH, random effect model was indicated. In addition, subgroup analysis and meta-regression were computed to estimate the pooled prevalence of LBW among women who had PIH and to identify potential source of heterogeneity using different study characteristics.

### Subgroup analysis

As stated above, this systematic review showed significant heterogeneity across included studies. So, subgroup analysis was done using region, publication year, study design, sampling technique and type of PIH. Regarding to regional prevalence, in Addis Ababa (AA) almost half of women who had PIH delivered LBW newborns (48.5 95% CI: 39, 57). Whereas in Amhara and SNNP, more than one third of newborns born from women who had PIH were LBW (39.6 95% CI: 26, 42 and 39.7% (95% CI: 22, 56 respectively). In Oromo region, 30.5% (95% CI: 23, 57) of newborns born from women with PIH experienced LBW.

Regarding to study design, studies done using case control design reported more than half of newborn babies born from women who had PIH had LBW (52.5%: 95% CI: 44, 60). While studies done in cohort and cross sectional indicated 42.59% (95% CI: 32, 52) and 35.44% (95% CI: 27, 43) of pooled prevalence of LBW among PIH women respectively. Ones more, studies which evaluate effect on PIH as general on LBW reported pooled prevalence of 39.03% (95% CI: 32, 45) and studies which taken effect of eclampsia on LBW indicated 45% (95% CI: 26, 63) of pooled prevalence of LBW (Table 2).

**Table 2:** Sub group analysis which describes pooled prevalence of LBW among women who had PIH with different study characteristics in Ethiopia: 2005–2020

Subgroup		No of studies	prevalence (95%CI)	Heterogeneity statistics	I^2^	*p*-value
Region	Amhara	8	39.64 (26,52)	197.34	96.5	< 0.001
	AA	4	48.56 (39,57)	17.99	83.3	< 0.001
	SNNPR	6	39.74 (22,56)	192.71	97.4	< 0.001
	Oromo	4	30.58 (23,37)	13.29	77.4	0.004
	Others	2	41.41 (33,48)	2.08	52	0.149
Publication year	Before 2016	9	43.38 (33,52)	125.6	93.6	< 0.001
	2016 & above	14	36.95 (28,45)	314.13	95.9	< 0.001
	Un-published	1	49 (39,54)	0.0	–	–
Study design	Cross sectional	15	35.44 (27,43)	319.85	95.5	< 0.001
	Cohort	5	42.59 (32,52)	40.59	90.1	< 0.001
	Case control	4	52.51 (44,60)	11.35	73.6	0.010
Sampling technique	Consecutive	16	37.60 (29,45)	336.15	94.6	< 0.001
	Systematic sampling	6	44.29 (33,53)	67.56	92.6	< 0.001
	Simple random	2	43.70 (7,80)	50.95	98	< 0.001
Type of hypertension	PIH	16	39.03 (32,45)	434.68	95.4	< 0.001
	Eclampsia	3	45 (26,63)	39.06	94.9	< 0.001

### Meta regression

As we have mentioned before, the forest plot detected statistically significant heterogeneity. So, in order to identify those potential source of heterogeneity, meta-regression was done using both continuous and categorical study characteristics: including publication year, sample size, mean age of the women, region, type of PIH, study design and technique. But, none of these variables were found to be statistically significant as shown below (Table 3).

**Table 3:** Meta regression for the included studies to identify source of heterogeneity for the pooled prevalence of LBW among women who had PIH in Ethiopia from 2005 to 2020

Variables	Coefficients	*p*-value
Publication year	−3.055	0.226
Sample size	−0.0073	0.748
Mean age	−1.637	0.743
Region		
AA	7.13	0.589
Amhara	−2.17	0.858
Oromo	−10.30	0.437
SNNPR	−3.44	0.784
Type study design		
Cohort	−8.93	0.358
Cross sectional	−18.54	0.330
Type sampling technique		
Survey	−5.32	0.672
Systematic random	1.39	0.918
Pregnancy induced hypertension	−9.18	0.351

**Fig. 3:**
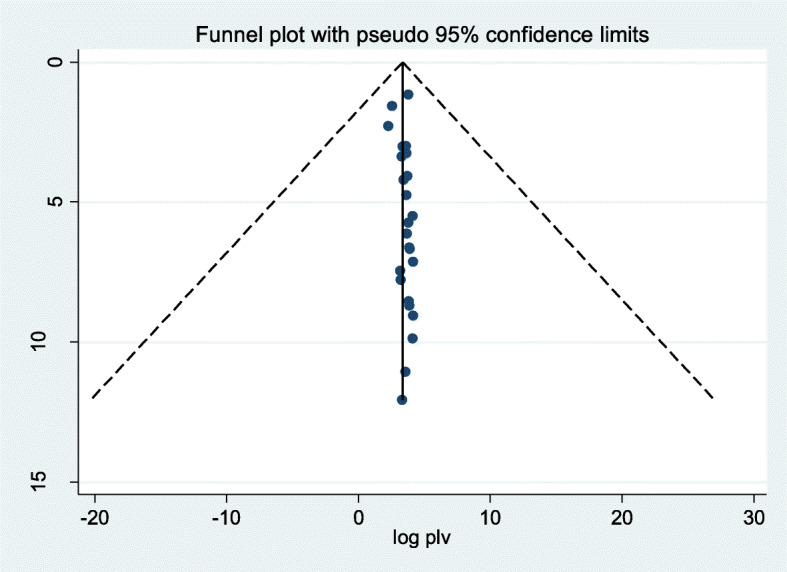
Meta funnel presentation of the pooled prevalence of LBW among women who had PIH in Ethiopia: 2005–2020

Furthermore, symmetric funnel plot and Egger’s tests were undertaken to assess publication bias. All of them confirmed publication bias was not observed, evidenced with the following symmetric funnel plot (Fig. 3), Egger’s test *p*-value =0.304 (95% CI: − 0.061, 0.186).

### The impact of pregnancy induced hypertension on low birth weight

**Fig. 4:**
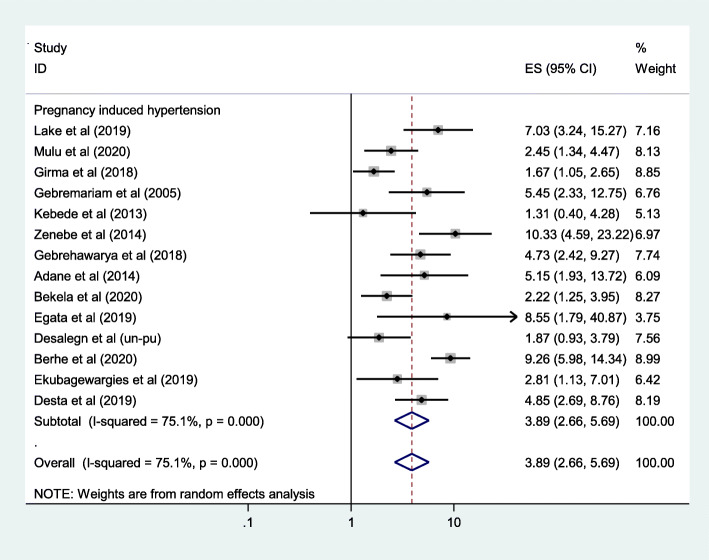
Forest plots which describe association between LBW and PIH in Ethiopia: 2005–2020

In addition to estimating the pooled prevalence of LBW among women who had PIH, this meta analysis also evaluate the odds of association between LBW and PIH. Accordingly, the odds of LBW among women who had diagnosed PIH was 3.89 times higher when compared with normotensive women (OR = 3.89, 95% CI: 2.66, 5.69) (Fig. 4).

## Discussion

Generally, the main aim of this systematic review and meta-analysis was to estimate the pooled prevalence of LBW among women who had PIH and to evaluate the impact of PIH on LBW in Ethiopia. Reduction of neonatal mortality is one of the major Sustainable Developmental Goals needed to be achieved by 2030 worldwide. But, neonatal mortality is still unacceptably high. Specifically in Ethiopia, the Mini Ethiopian Demographic Health Survey 2019 reported that neonatal mortality was still sustained [59] and LBW is one of the major causes of neonatal mortality. So, in a country like Ethiopia where striving to reduce neonatal mortality in 2030, investigating such concrete scientific evidence will be used as indicator for maternal and child health cares and input for health policy, programmers and implementers to improve neonatal health.

This review showed that, more than one-third of neonates born from women who had PIH were LBW. The pooled prevalence ranges from 33.3 to 46.2% with average estimate of 39.7%. This finding is in line with studies conducted in India [60], Uganda [61], Zimbabwe [62], Egypt [63] and systematic review done in Ethiopia [30]. This the finding is lower than studies done in Bangladesh [64], Tanzania [65] and Sudan [66]. However, it is higher than result of secondary analysis of the WHO multi-country survey done on 29 countries from Africa, Asia, Latin America and the Middle East [67]. In addition, this figure is almost two times higher than studies conducted in Malaysia [68] and Ghana [69]. This could be due to the difference in prevalence of PIH across countries and socioeconomic factors as well as attributed to difference in health facility characteristics such as service provision and diagnostics capability.

Furthermore, this figure is more than two times higher than the pooled estimate of LBW among the total population in Ethiopia [70]. The difference may be due to our review included only studies conducted among women who had PIH, one of the major risk factor for LBW. So, improving the availability, accessibility and quality of maternal health care services is warranted.

In regarding to the association between LBW and PIH, the odds of LBW among women who had PIH were higher when compared to normotensive women. This finding is in line with WHO secondary analysis survey conducted in low and middle income countries, which confirmed that the risk of acquiring LBW among women with PIH was double [71]. Similarly, studies conducted in United Kingdom [72], China [73] and Malaysia [73] reported that women who had PIH were at higher odds to have LBW newborns. This is also supported by studies held in Haiti [74] and Ghana [69], reported that women who had PIH were two times more likely to have LBW newborn babies. Many studies confirmed that, in PIH the trophoblast invasion in to the spiral arteries that supply the placenta is incomplete [75, 76]. Because of decreased utero-placental blood perfusion, leads to small for gestational age, preterm birth and intrauterine growth restriction which end up with LBW [76]. Therefore, provision of timely and effective care to the women presenting with these complications is essential.

Generally, even though still difficulties are there in middle and low income countries including Ethiopia, low dose aspirin and calcium supplementation, prenatal care, timely diagnosis, proper management, timely delivery and comprehensive intensive neonatal care service were very important. But, in Ethiopia, still three out of four women get antenatal care and less than half of them delivered at health facility [59]. So, improvement of maternal and child health services were warranted because PIH is not only causes neonatal morbidity and mortality but also maternal morbidity and mortality.

This systematic review and meta analysis is not without limitation. More than half of included articles were cross sectional study design in which the result might potentially affected by confounding variables and difficult to establish temporal relationship. In addition, the meta analysis didn’t include all regional states of Ethiopia. Finally, presence of significant statistical heterogeneity among studies was considered as limitation of this review. Therefore, further longitudinal country based studies to assess association of LBW and PIH is recommended.

## Conclusion

This systematic review and meta analysis revealed that, the pooled prevalence of LBW among women who had PIH was more than two times higher than the pooled estimate of low birth among general women. The odds of having LBW among women who had PIH was higher when compared with normotensive women. Even though prevention of PIH is difficult, early detection of women at high risk of having PIH coupled with intensive antenatal care and management could prevent the occurrence of this burden of LBW. In addition, improving the availability of antihypertensive therapy and intensive neonatal care service should be addressed. Therefore, enhancing universal access of comprehensive and quality perinatal care and obstetric emergency care services are needed to improve neonatal health in Ethiopia.

## Data Availability

Data will be available from the corresponding author upon reasonable request.

## Abbreviations

AA: Addis Ababa
LBW: Low Birth Weight
PIH: Pregnancy Induced Hypertension
SNNP: South Nation and Nationality peoples
WHO: World Health Organization
